# Mosquito transmission, growth phenotypes and the virulence of malaria parasites

**DOI:** 10.1186/1475-2875-12-440

**Published:** 2013-12-06

**Authors:** Laura C Pollitt, Margaret J Mackinnon, Nicole Mideo, Andrew F Read

**Affiliations:** 1 Center for Infectious Disease Dynamics, Millennium Science Complex, Pennsylvania State University, University Park, State college, Pennsylvania 16801, USA; 2 Kenya Medical Research Institute–Wellcome Trust Research Programme, Kilifi, Kenya; 3 Nuffield Department of Medicine, University of Oxford, Oxford, UK; 4 Department of Ecology and Evolutionary Biology, University of Toronto, Toronto, Canada; 5 Department of Entomology, The Pennsylvania State University, University Park, Pennsylvania, USA; 6 Fogarty International Center, National Institutes of Health, Bethesda, Maryland, USA

**Keywords:** Malaria, Transmission, Virulence, Mosquitoes, Within-host dynamics, *Plasmodium chabaudi*

## Abstract

**Background:**

A series of elegant experiments was recently published which demonstrated that transmission of malaria parasites through mosquitoes elicited an attenuated growth phenotype, whereby infections grew more slowly and reached peak parasitaemia at least five-fold lower than parasites which had not been mosquito transmitted. To assess the implications of these results it is essential to understand whether the attenuated infection phenotype is a general phenomenon across parasites genotypes and conditions.

**Methods:**

Using previously published data, the impact of mosquito transmission on parasite growth rates and virulence of six *Plasmodium chabaudi* lines was analysed.

**Results:**

The effect of mosquito transmission varied among strains, but did not lead to pronounced or consistent reductions in parasite growth rate.

**Conclusions:**

Mosquito-induced attenuated growth phenotype is sensitive to experimental conditions.

## Background

Malaria infections vary hugely in the amount of harm caused to their hosts (virulence). This variation can, in part, be explained by host factors but parasites themselves also vary [1-4]. In a recent publication, Spence *et al.*[5] demonstrated that the virulence of an infection may be mediated by the environment that parasites have experienced. Using a model rodent malaria system, they showed that malaria parasites are intrinsically modified during the process of developing within the vector and establishing a new infection in a vertebrate host. Parasites that had recently been transmitted through a mosquito displayed an attenuated growth phenotype, with infections growing more slowly and achieving a peak parasitaemia at least five-fold lower than that achieved by parasites that had not been mosquito transmitted (MT). Spence *et al.*[5] demonstrated that this altered phenotype was correlated with the expression of a different set of antigenic proteins in the parasites eliciting a different immune response in the host, to which they attribute the enhanced control of parasite replication. The attenuated growth phenotype was associated with less severe virulence, including reduced anaemia and weight loss.

**Figure 1:**
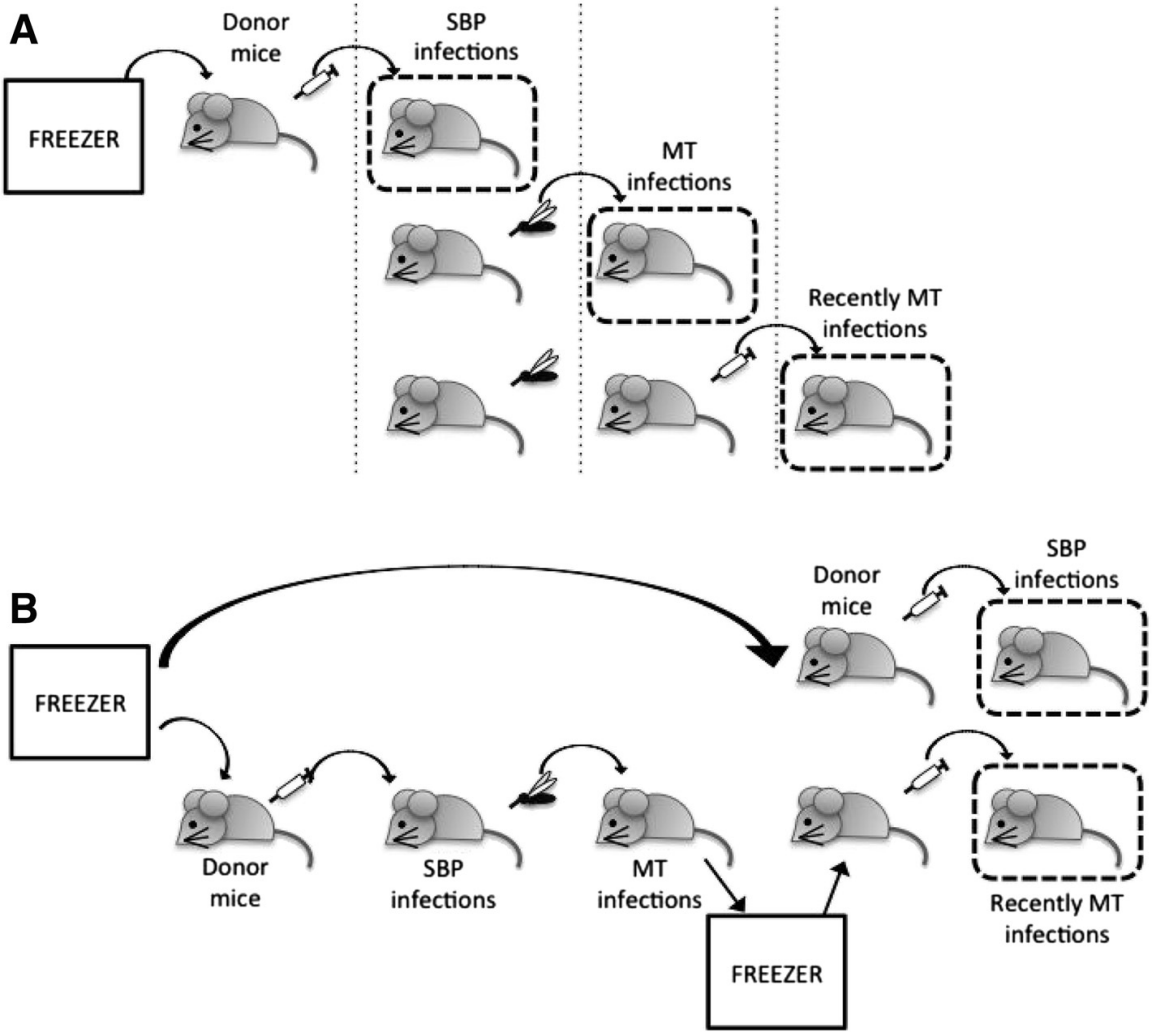
**Comparison of infection protocol between (A) Spence *et al. ***[5]**and (B) Mackinnon *et al. ***[6]**.** Dashed boxes indicate the hosts from which data for the different types of infections (SBP, serially blood-passaged; MT, mosquito transmitted; recently MT) were generated.

Their study has two important implications. Firstly, demonstrating the effect of parasite expression profiles on host immunity and disease progression sheds light on the mechanisms of protective immunity in malaria infections. Secondly, these results call into question the standard methodology used in model rodent malaria systems. For convenience, and because it is the blood stage infection which causes disease, the majority of laboratory studies examining the interactions between malaria parasites and their host bypass the vector and initiate infections with direct serial passage of blood stage parasites. The study by Spence *et al.*[5] suggests that this non-natural route of infection may alter parasite phenotypes and infection outcomes. To assess the implications of these results for future research in this area, understanding whether the attenuated infection phenotype is a general phenomenon across a broader range of parasites genotypes and conditions is crucial. This paper seeks to address this question by reanalysing a previously published dataset of parasite densities and measures of virulence from experimental malaria infections initiated with parasites that differed in their transmission histories. The experimental design differs in subtle ways from that of Spence *et al.*[5] (Figure 1), but this is the best existing dataset for the task.

## Methods

Full experimental design and parasite line histories are reported elsewhere [6] (see also Figure 1B). Briefly, six lines of *Plasmodium chabaudi* (CW-0, CW-A, CW-V, ER, AS, AJ) were either transmitted though mosquitoes or maintained via serial blood transfer. Three lines that shared an ancestral genotype had different passage histories (CW-O: recently derived from a wild isolate; CW-A, CW-V: multiple passages through mice). All infections were initiated via IP injection with 10^4^-10^6^ blood stage parasites and parasitaemia, mouse weight and erythrocyte density (anaemia) were monitored.

All analysis was performed using R (version 2.14.1). Day 8 parasitaemia (proportion of red cells infected with malaria parasites) was analysed using a general linear model with binomial errors. Day 8 parasite density (number of parasites per microlitre of blood) was log transformed to fit models assumptions before analysis with a general linear model. Model simplification was followed by sequentially dropping the least significant term and comparing the change in deviance, with and without the term.

## Results and discussion

**Figure 2:**
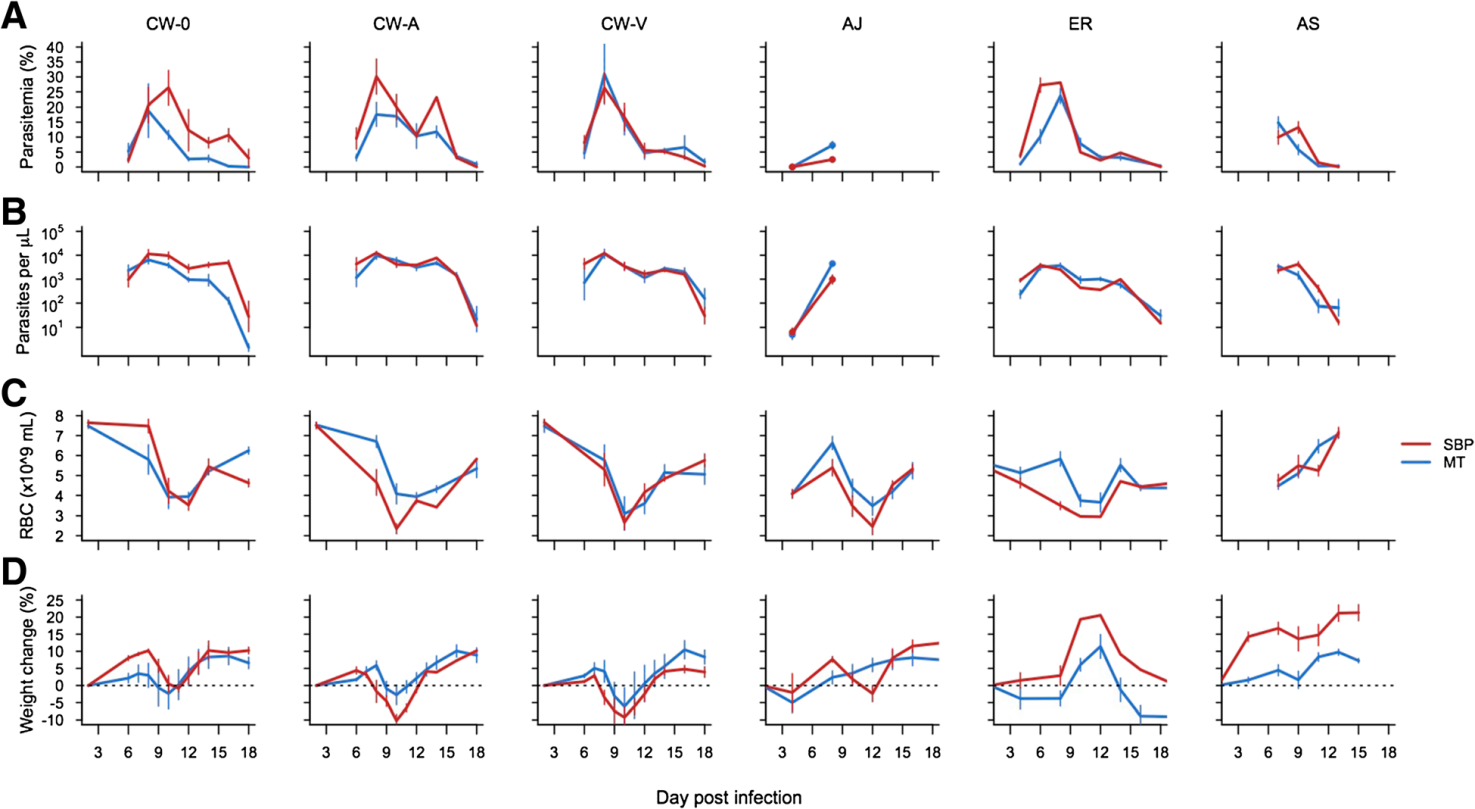
**The effect of mosquito transmission on infection dynamics and virulence depends on parasite line.** Graphs show **A)** parasitaemia, **B)** parasite density, **C)** red blood cell density, **D)** change in weight from time of infection. Means of five to six replicate infections for serially blood-passaged parasites (red) and recently mosquito-transmitted lines (blue). Parasite line is shown at the top of each column. Bars show the standard error of the mean.

Spence *et al.* focussed on one line of *Plasmodium chabaudi* (AS) although a second line (CB) showed qualitatively similar patterns [5]. In Figure 2, data is shown for six parasite lines (four unique genotypes) including an AS line from the same wild-type ancestor as used by Spence *et al*. [3]. The effect of recent mosquito transmission on parasitaemia and parasite density was inconsistent among parasite lines (parasite line* transmission route interaction on day 8 parasitaemia (χ^2^_5,49_, p < 0.001) and on day 8 parasite density (F_5,49_ = 4.01, p = 0.004)). Where mosquito transmission did reduce growth, the reduction was substantially less pronounced (maximum 1.5-fold) than the > five-fold differences reported by Spence *et al.* (compare relative peak parasitaemias in Figure 2A with those in Spence *et al.* Figures 1A and B). PCR data on early growth rates (days 4–7) confirmed this lack of difference in parasite replication rates [6].

Two measures of virulence, anaemia and weight loss, were measured. In the experiments of Spence *et al.*, the attenuated growth phenotype was associated with negligible weight loss, and a doubling of minimum red blood cell (RBC) densities compared to infections initiated with serial blood passage (SBP); in the experiments reported here, mosquito transmission, on average, reduced weight loss and anaemia during infection, but the effect was relatively minor and varied among strains ([6] and Figure 2C and D).

There are likely many small differences in the experimental set up in the two studies. For instance, the strain of the vector (although not the mice) varied between studies. Mouse diet also varied: the mice used here [6] were provided with para-aminobenzoic acid (PABA) which is known to increase parasite growth rates [7]. Since the patterns of parasitaemia from SBP initiated infections presented here do not differ substantially from those in [5], whether or not PABA could have a differential affect on SBP and MT parasites remains an open question. More subtle, but potentially important, is a difference in the timing of infections in the two studies. The infection protocols are illustrated in Figure 1. In both cases, data for SBP initiated infections are generated by injecting parasitized RBCs into experimental hosts, after growing up parasites from cryopreserved stocks in donor mice, which can involve multiple passages in different donor mice before experimental infections. Spence *et al.* initiated MT infections directly from such SBP-infected mice, allowing them to compare infections initiated before and directly after the transmission event. Recently MT infections – those generated by injecting parasitized RBCs from MT infections into new hosts – also showed the attenuated phenotype.

This latter group of infections most closely resembles the mosquito-transmitted lines from Mackinnon *et al.*[6], reported here. Blood from mosquito-transmitted infections was cryogenically preserved (so that SBP and MT infections could be compared contemporaneously). Parasites were subsequently grown up from these samples in donor mice and injected into experimental hosts. ‘Recently MT’ parasites were serially blood-passaged between two and five times (Figure 1 in [6]) compared to one round of SBP in Spence *et al.*’s recently MT parasites. Although Spence *et al.* did not explore the dynamics of infection with recently MT parasites that endured more rounds of SBP, they showed that the attenuation of day 7 parasitaemia generated from MT starts to degrade after five rounds (Supplementary Figure Five in [5]). From these data, it would be expected that the MT lines from Mackinnon *et al.* would still demonstrate an attenuated phenotype, particularly the ER and the three CW lines, which were passaged only twice. Whether freezing, which the MT lines analysed here experienced, fundamentally alters antigen expression and virulence is an important open question, of interest to all researchers using cryopreserved malaria parasite stocks.

Finally, differences in pre-mosquito passage history may be important. The AS line used by Spence *et al.* had undergone SBP 26–30 times since isolation from the natural host; the lines shown here been passaged a maximum of 11 time [6]. The three CW lines reported here also share a common ancestor, but have different pre-transmission passage histories - the line with the greatest number of passages showed the smallest effect of mosquito transmission (CW-V; Figure 2) in this and another experiment [8].

Clearly, all of the above hypotheses can be tested empirically. The re-analysis of earlier experiments has been reported here to encourage such further work and so that those following up the important work of Spence *et al.* are aware that experimental details apparently matter considerably.

## Conclusions

Spence *et al*. suggested that the growth rate attenuation they discovered is the result of differences in antigenic expression profiles, regulated by something in the mosquito transmission pathway, which elicit different host immune responses. Here it is shown that, in earlier experiments, mosquito passage did not consistently or substantially attenuate parasite growth phenotype. The experiments from which these data arose differ from the experiments of Spence *et al*. in a number of potentially important ways. This suggests that a comparison of host immune profiles generated by infections with a number of different lines may shed more light on the strain variation and qualitative differences in the effects of mosquito transmission. Whether such alteration of expression profiles is, for example, vector or parasite strain specific is unknown.

## Competing interests

The authors declare they have no competing interests.

## Authors’ contributions

MJM conducted the original experiments from which data presented here arose and LCP performed the new analysis. All authors contributed to drafting the manuscript. All authors read and approved the final manuscript.
